# Determinants of practice of preconception care among women of reproductive age group in southern Ethiopia, 2020: content analysis

**DOI:** 10.1186/s12978-021-01154-3

**Published:** 2021-05-21

**Authors:** Aklilu Habte, Samuel Dessu, Dereje Haile

**Affiliations:** 1 Department of Public Health, College of Medicine and Health Sciences, Wachemo University, Hosanna, Ethiopia; 2 Department of Public Health, College of Medicine and Health Sciences, Wolkite University, Wolkite, Ethiopia; 3 Department of Reproductive Health and Nutrition, School of Public Health, College of Medicine and Health Sciences, Wolaita Soddo University, Soddo, Ethiopia

**Keywords:** Preconception care, Contents of care, Determinants, Ethiopia

## Abstract

**Background:**

Preconception care (PCC) is a series of biomedical, mental, and psycho-social health services provided to women and a couple before pregnancy and throughout subsequent pregnancies for desired outcomes. Millions of women and new-borns have died in low-income countries due to impediments that arise before and exaggerate during pregnancies that are not deal with as part of pre-conception care. To the best of our knowledge, however, there is a lack of information about preconception care practice and its determinants in southern Ethiopia, including the study area. This study was therefore planned to assess the practice of preconception care and its determinants among mothers who recently gave birth in Wolkite town, southern Ethiopia, in 2020.

**Methods:**

A community-based cross-sectional study was conducted from February 1 to 30, 2020. A total of 600 mothers who have given birth in the last 12months have been randomly selected. A two-stage sampling technique was employed. For data collection, a pre-tested, semi-structured questionnaire was used. The data was encoded and entered into Epi-Data version 3.1 and exported for analysis to SPSS version 23. Household wealth status was determined through the application of principal component analysis(PCA). The practice PCC was considered as a count variable and measured as a minimum score of 0 and a maximum of 10. A bivariable statistical analysis was performed through analysis of variance (ANOVA) and independent t-tests and variables with a p-value of<0.05 were eligible for the generalized linear regression model. To see the weight of each explanatory variable on PCC utilization, generalized linear regression with a Poisson link was done.

**Results:**

Of the sampled 600 participants, 591 took part in the study, which yielded a response rate of 98.8%.The mean (SD) score of the practice of PCC was 3.94 (1.98) with minimum and maximum scores of 0 and 10 respectively. Only 6.4% (95%CI: 4.6, 8.6) of mothers received all selected items of PCC services. Thecommonest item received by 67.2% of mothers was Folic acid supplementation, while 16.1% of mothers received the least item of optimizing psychological health. Education status of mother[AOR 0.74, 95%CI 0.63, 0.97], time spent to access nearby health facilities [AOR 0.69, 95%CI 0.58, 0.83], availability of PCC unit [AOR1.46; 95%CI 1.17, 1.67], mothers knowledge on PCC [AOR 1.34, 95%CI 1.13, 1.65], being a model household [AOR 1.31, 95%CI 1.18, 1.52] and womens autonomy in decision making [AOR 0.75, 95%CI 0.64, 0.96] were identified as significant predictors of practice of PCC.

**Conclusion:**

The uptake of WHO-recommended PCC service elements in the current study area was found to be unsatisfactory. Stakeholders must therefore increase their efforts to align PCC units with existing MNCH service delivery points, improve women's decision-making autonomy, and focus on behavioral change communication to strengthen PCC practice.

**Plain language summary**

Preconception care (PCC) is a series of biomedical, mental, and psycho-social health services provided to women and a couple before pregnancy and throughout subsequent pregnancies for better endings. The main goal of the PCC is to improve maternal and child health outcomes, by-promoting wellness and providing preventive care. It can also be seen as an earlier chance for teenage girls, mothers, and children to live a better and longer-term healthy life. Pieces of PCC service packages suggested by the World Health Organization(WHO) are, micronutrient supplementation (Folate supplementation), infectious disease (STI/HIV) screening and testing, chronic disease screening and management, healthy diet therapy, vaccination, prevention of substance use (cessation of cigarette smoking and too much alcohol consumption), optimizing psychological health, counseling on the importance of exercise and reproductive health planning and implementation. Millions of women and new-borns have died in low-income countries due to impediments that arise before and exaggerate during pregnancies that are not deal with as part of pre-conception care. To the best of our knowledge, however, there is a lack of information about preconception care practice and its determinants in southern Ethiopia, including the study area. This study was therefore planned to evaluate the practice of preconception care and its determinants among mothers who recently gave birth in Wolkite town, southern Ethiopia, in 2020.

Mothers who have given birth in the last 12months have been randomly selected Household wealth status was determined through the application of principal component analysis(PCA). To see the weight of each explanatory variable on PCC, generalized linear regression with a Poisson type was done. Accordingly, the Education status of the mother, time spent to access nearby health facilities, availability of PCC unit, mothers knowledge on PCC, being a model household, and womens autonomy in decision making were identified as significant predictors of practice of PCC. Stakeholders must therefore increase their efforts to align PCC units with existing MNCH service delivery points, improve women's decision-making autonomy, and focus on behavioral change communication to strengthen PCC practice.

## Background

Preconception care (PCC) is a series of biomedical, mental, and psycho-social health services offered to women and a couple before pregnancy and throughout subsequent pregnancies [1, 2]. It is the earliest connection between maternal and infant health and a window of opportunity to reinforce the health of women before, during, and after pregnancy through early detection and risk management [3, 4]. It is a part of the Healthy People initiative that focuses profoundly on tackling unwanted pregnancies [5]. The main goal of PCC is to improve maternal and child health outcomes by promoting wellness and providing preventive care. It can also be seen as an earlier opportunity for adolescent girls, mothers, and children to live a better and longer-term healthy life [6, 7].

Preconception care offers a variety of services in the sense of prevention aimed at mothers and newborns [8, 9]. Several pieces of PCC service packages were suggested by the World Health Organization(WHO), including micronutrient supplementation (ferrous supplementation), infectious disease (STI/HIV) screening and testing, chronic disease screening and management, healthy diet therapy, vaccination, prevention of substance use (cessation of cigarette smoking and too much alcohol consumption), optimizing psychological health, counseling on the importance of exercise and reproductive health planning and implementation [1, 10, 11].

Pregnancy and childbirth complications resulted in the deaths of 830 and 303,000 women daily and annually, respectively, as well as a great deal of suffering and long-term disabilities [12]. More than 3.1 million newborns died in their first month of life around the world, with 14.9 million born prematurely and 2.7 million stillborn [13]. According to the Ethiopian Demographic Health Survey (EDHS) 2016 report, the pregnancy-related mortality ratio was 412 per 100,000 live births and the lifetime risk of pregnancy-related death is 21 in 1000 women [14]. Most of these complications happen before and get worse during pregnancy, especially if they are not treated as part of PCC [15].

The Sustainable Development Goal (SDG) set a goal of minimizing the ratio of maternal mortality below 70 per 100,000 live births and the rate of newborn mortality (NMR) to 12 per 1000 live births and s globally in 2030 [16]. PCC helps in achieving this goal by reducing adverse birth outcomes like preterm birth, low birth weight, and congenital defects by expanding access to high-quality PCC service packages [17,19].

PCC is among the key programs in the continuum of care(COC) for maternal neonatal and child health (MNCH) care [20, 21]. According to the recommendation of the Center for Disease Control (CDC), every childbearing woman should obtain a risk assessment during her primary health care visit to realize good pregnancy and maternity outcomes [22]. In contrast, millions of women all around the globe do not have access to pre-conception service packages, especially in low-income countries [23]. Recent reports have shown that approximately 40% of global women have undergone unplanned pregnancies, suggesting that 4 out of 10 women do not have vital health intervention packages before conception [16, 24].

Studies conducted in Utah, Brazil, China, Sri Lanka, and Saudi Arabia, have shown a low level of PCC service uptake of 15.932.1% for women of reproductive age [25,29]. Studies conducted in Ethiopia have shown that the practice of PCC necessitates further enhancement with a magnitude of 9.638.2% [30,34]. Factors such as maternal age, level of education, occupational status, prior history of adverse birth outcomes, pregnancy planning status, PCC awareness, and PCC unit availability have been identified as possible deteminats [30,33].

While the PCC is one of Healthy People 2020's major strategic objectives, it has not been extensively understood and practiced, especially in low-income countries, including Ethiopia [6, 35, 36]. In contrast with other Continuum of Care (COC) maternal and child health services, the PCC can be viewed as the missing continuum elements [37]. Besides, the CDC renowned that PCC packages are not acknowledged by most people and would need more intervention [7, 38].

Although the Ethiopian government has given due importance over the last decades to maternal and child health services in terms of prenatal care, skilled delivery service, and postpartum care, adverse pregnancy outcomes and congenital defects are-at a frightening rate [39]. These adverse pregnancy outcomes will raise the risk of noncommunicable diseases like cardiovascular disease and other chronic diseases in both mothers and children, thereby promoting transgenerational disease [38]. In recent decades, good accomplishments have been noted in the overall prenatal care service, but the median month for the first ANC visit is 4.7months [14]. This late ANC initiation can be reduced by providing adequate pregnancy preparation through the integration of PCC with MNCH services [1, 8, 38]. PCC service packages are currently available on a range of service delivery channels, but there are still many shortcomings in providing the service on a daily and regular basis [30, 32, 33, 39]. Little is known, including the current study area, about the practice of PCC and its determinants among childbearing women in Wolkite town, southern Ethiopia.

Assessing the level of PCC experience and its determinants allows policymakers to foresee the PCC needs of women and couples before pregnancy, ensuring that motherhood and childhood are off to the safest start possible. This research was therefore intended to examine the practice of PCC and to recognize determinants. Also, the research will contribute to the creation and implementation of successful strategies to increase service adoption and, ultimately, to resolve the increasing burden of pregnancy and adverse outcomes.

## Materials and methods

### Study design and settings

The Community-based cross-sectional study was conducted from 1 to 30 February 2020 in Wolkite town, southern Ethiopia. The town is located 151km southeast of the capital city, Addis Ababa. Administratively, the town is divided into 13 kebeles (*Kebele is the smallest administrative unit in Ethiopia*). The projected total population of the town for the 2020 fiscal year was 102,948 (Male=55,858, Female=52,090), based on the 2007 CSA forecast. The total number of women in the reproductive age group (1549years) was 23,246, representing 22.6% of the population as a whole. In the town that was providing maternal and child health services, there are a total of 19 health facilities and further quantified as one teaching and referral hospital, two health centers, 10 health posts, and six medium clinics.

### The population of the study

The source populations for this study were all mothers in the town who had given birth in the preceding 12months. The study population is made up of women in seven randomly selected kebeles in the town who come across eligibility criteria. Women who had lived in the study area for less than 6 months and were critically ill at the time of data collection were excluded from the study.

### Sample size determination

The sample size for the study was determined by applying the single population proportion formula. The parameters such as the reported prevalence of PCC practice, which is 38.2% from a study conducted in western Ethiopia [34], a 95% confidence level, 5% margins of error, 1.5% design effect, and 10% non-response rate was used. Consequently, the final sample size for the study was 600.

### Sampling techniques

To get study participants, a two-stage sampling technique was used. There are 13 Kebeles in the town, and seven of them were chosen randomly by lottery method. The total number of mothers who had given birth in the previous 12months in each kebele was taken from the delivery registration books located in health posts. The sample size for each kebele was distributed by proportional allocation and each study participant was accessed by using the lottery method. When more than one eligible woman was identified in the chosen household, a lottery method was applied.

### Data collection tools, techniques, and personnel

Six diploma nurses with previous data collection experience conducted face-to-face interviews under the supervision of three BSc Midwives, using a pre-tested, standardized questionnaire. Both data collectors and supervisors were given intense training that lasted one day. An intensive training that lasts one day was provided to both data collectors and supervisors. The data collection tool was developed after reviewing relevant literature in the field of interest [1, 8, 31, 33]. The questionnaire contains socio-demographic variables, obstetric variables, health system-related features, and respondents' knowledge of PCC. The wealth status of households was evaluated using a standardized questionnaire adapted from EDHS 2016 [14]. The pre-test was conducted prior to the actual data collection. Women were contacted with the guidance of local community health workers (CHWs) in each Kebele. The list of women selected for the interview in each kebele was provided in advance to the data collectors. The data were collected on a house-to-house basis.

### Data quality management

The questionnaire used to collect data was initially prepared in English, then translated into the local language by an expert in that language, and then re-translated into English to ensure consistency with the original meanings. One-day intensive training was provided to both data collectors and supervisors on the purpose of the study, the content of the questionnaire, and the method of data collection was. A pretest among 5% of the sample size (30 respondents) was conducted in Wolliso town, and possible adjustments were made for the better completion of the questionnaire. A day-to-day follow-up during the data collection period was carried out by the principal investigator and supervisors. Before entering the data, the supervisors checked it for completeness and consistency. Incomplete data were omitted from the analysis. To reduce social desirability bias, study participants were interviewed privately.

### Data analysis

The data was coded and entered into Epi Data Version 3.1 and exported to SPSS version 23 for analysis. Descriptive statistics such as frequencies, mean, and standard deviation were computed to describe the characteristics of the respondents. The wealth status of households was determined by applying principal component analysis (PCA). Initially, 27 items were used and grouped into six components, namely: household properties, livestock ownership, crop production in quintal, the average estimated monthly income, hectares of agricultural land, and housing conditions. Fulfillment of PCA assumptions such as; overall sampling adequacy (KMO0.6), sampling adequacy of individual variables (anti-image correlations>0.4), Bartlett Sphericity Test (p-value<0.05) were checked. In each step, these variables with communities less than 0.5 and complex structures (i.e. having correlations greater than 0.4 in more than one component) were removed before the criteria were met iteratively. Finally, three components were extracted from the PCA that clarified a total variance of 66.94% and used to rate the study participants' household wealth status in quintiles [14].

A bivariable analysis using analysis of variance (ANOVA) and independent t-tests had been used to test statistical significance, and variables with a p-value of 0.05 were eligible for a generalized linear regression model. Multivariable statistical analysis using the Generalized Linear Model (GLM) approach was used to classify the determinants of PCC practice. A Poisson regression model with a log link was used since our response variable was measured in terms of count variables [40]. The property of equidispersion, which states that the variance of a distribution of count-dependent variables is equal to its mean, is one of the basic assumptions of Poisson regression [40, 41], and the current study almost meets this criterion with mean and variance of 3.940 and 3.938, respectively. Finally, the odds ratios and 95% confidence intervals for each independent variable were calculated. The strength and direction of the association were determined using crude and adjusted odds ratios. Charts, graphs, and figures were used to display the information.

### Variables of the study

#### Dependent variable

Preconception care: the complete range of interventions to promote the well-being of the expectant mother and the baby [1]. The ten selected items of PCC services considered in this study were: Folic acid supplementation, vaccination, screening and management of infectious diseases (STI/HIV), screening and management of chronic diseases, balanced diet therapy, cessation of cigarette smoking, avoidance of excessive alcohol consumption, optimization of psychological wellbeing, provision of modern contraceptive/s [1, 8]. Information on these ten contents of PCC was derived from the response to the question Prior to your last pregnancy, did you received any of the following services at least once? Have you got any vaccination?, Have you got any contraceptive? Answer categories were developed for each practice assessment question as' YES=1' and' No=0. It is possible for a single mother may get a vaccination or modern contraceptive several times prior to pregnancy. However, as the mother was asked to report any service at least once, the response for any action was recorded as a single action. On the basis of responses, we have created a composite index of PCC content as our outcome variable which comprises a simple count of the number of elements of care received. The variable had a minimum value of zero indicating that the women did not receive any PCC services and a maximum value of ten indicating that the women received services for all the ten elements.

#### Explanatory variables

Household wealth index: a composite measure of respondents' socio-economic status was computed using PCA based on data from sustainable household goods and equipment, livestock ownership, quintal crop production, the average projected monthly income, agricultural land in hectares, and residential housing characteristics. Finally, the first component which explained maximum factor scores were split into quintiles [14].

Women's Autonomy in household decision-making: A woman is said to be autonomous of decision-making power when she decides on at least one of the following three issues alone or jointly (with her husband): (1) the health of the woman (personal decision-making authority), (2) big transactions (economic decision-making authority), and (3) visits to friends or relatives (mobility decision-making authority)otherwise considered as non-autonomous when the husband alone or a third person decides on seeking MNCH services [14, 42, 43].

Knowledge on PCC:: If a woman correctly answered at least 50% of the correct answers to eight PCC knowledge assessment questions, she was classified as knowledgeable; otherwise, she was classified as not knowledgeable [31, 44].

Being a household model (MHH): a family that implements all health extension packages and has received certificates of appreciation from responsible bodies [45].

Perceived distance to the nearest health facility: This was determined by the respondents' answers to questions of how long they walked to the health facilities. If mothers reported walking for less than 30min to reach the nearest health facility, this was coded as 'closer;' otherwise, it was coded as 'far' [46].

Preconception care unit: This is a unit or space where preconception care for women was offered before becoming pregnant [31].

## Results

The study included a total of 591 mothers who gave birth within the last 12months, which resulted in a 98.9**%** response rate.

### Socio-demographic characteristics of respondents

The age of respondents ranges from 16 to 44years with a mean age of 27.9 and with a standard deviation of5.4years. The majority of respondents were married (86.3%) and more than one-third (36.2%) had attended primary education. The predominant ethnic group was Guraghe (87.4%) and almost half (47.7%) were Orthodox by religion (Table 1).

**Table 1:** Distribution of Socio-demographic characteristics of study participants in Wolkite town, Southern Ethiopia, February 115, 2020

Variables categories	Frequency	Percent
Age (n=591)		
<24	218	36.9
2534	289	48.9
35+	84	14.2
Marital status(n=591)		
Married	510	86.3
Divorced	40	6.8
Widowed	24	4.1
Single/never married	17	2.8
Ethnicity (n=591)		
Guraghe	510	86.3
Amhara	66	11.2
Others*	15	2.5
Religion(n=591)		
Orthodox	282	47.7
Muslim	239	40.4
Protestant	64	10.8
Catholic	6	1.1
Mothers education level(n=591)		
No formal education	112	19.0
Primary education	214	36.2
Secondary education	182	30.8
College and above	83	14.0
Mothers occupation (n=591)		
Housewife	235	39.8
Private business work	183	31.0
Government employer	130	22.0
Student	43	7.2
Husbands education level (n=510)		
No formal education	102	20.1
Primary education	151	29.6
Secondary education	167	32.7
College and above	90	17.6
Husband occupation (n=510)		
Merchant	249	48.8
Farmer	114	22.4
Government employer	103	20.2
Daily laborer	44	8.6
Family size (n=591)		
>5	145	24.5
<5	446	75.5
Household wealth status(n=591)		
Poorest	106	17.9
Poorer	112	19.0
Middle	131	22.1
Rich	125	21.2
Richest	117	19.8

*Other ethnicity=Oromo, Tigri

### Obstetric characteristics of respondents

The obstetric characteristic assessment found that nearly half (49.6%) of participants were multiparous. 'Majority of the participants, 536 (85.8%) visited health facilities for ANC service at least once for their recent pregnancy and one-third (33.2%) of them got four or more visits. More than half (328, 55.5%) of respondents used modern family planning, and there were 138 (23.3%) among those existing users. One hundred forty-nine (25.2%) of study participants reported a prior history of adverse birth outcomes, in which 45 (30.2%) and 33 (22.1%) of women reported a history of spontaneous abortion and stillbirth, respectively (Table 2).

**Table 2:** Obstetric characteristics of respondents who gave birth within the last 12months in Wolkite town, Southern Ethiopia, February 130, 2020

Variable categories	Frequency(n)	Percentage
Parity(N=591)		
Primipara	122	20.6
Multipara	293	49.6
Grand multipara	176	29.8
Modern FP utilization(N=591)		
Yes	328	55.5
No	263	45.5
FP by method mix (n=591)		
Oral contraceptives	108	32.9
Injectable	130	39.6
Implants	55	16.8
IUD	35	10.7
ANC attendance		
Yes	528	89.3
No	63	10.7
Frequency of ANC visit (n=528)		
One visit	59	11.2
Two visits	91	17.2
Three visits	185	35.1
Four and more visits	193	36.5
Place of delivery service(N=591)		
Health center	445	75.3
Hospital	110	18.6
Health post	24	4.1
Home	12	2.0
Having a History of adverse birth outcome (N=591)		
Yes	149	25.2
No	442	74.8
Types of adverse birth outcome (N=149)		
Spontaneous abortion	45	30.2
Preterm birth	32	21.5
Stillbirth	33	22.1
Neonatal death	25	16.8
Congenital anomalies	14	9.4
History of unintended pregnancy (N=591)		
Yes	98	16.6
No	493	83.4

### Health system-related characteristics of the respondents

Concerning the accessibility of health services, the majority (79.7%) of mothers have access to health facilities within fewer than 30min and more than half (56.5%) use the foot as a means of transport. The availability of adequate medication and laboratory facilities nearby was recorded by almost three-fifths (58.2%) and more than half (53.6%) of the participants respectively. The availability of PCC guidelines was mentioned by nearly one-third of respondents, 193 (32.6%). For decision-making autonomy, the majority of participants (84.4%) were autonomous (Table 3).

**Table 3:** Health services related characteristics of respondents in Wolkite town, Southern Ethiopia, 130 February 202

Variables categories	Frequency	Percent
Availability of adequate laboratory service(n=591)		
Yes	317	53.6
No	239	40.4
I dont know	35	5.9
Availability of adequate medication(n=591)		
Yes	344	58.2
No	219	37.1
I dont know	28	4.7
Availability of PCC unit(n=591)		
Yes	239	40.4
No	321	54.3
I dont know	31	5.2
Availability of Guideline(n=591)		
Yes	193	32.6
No	398	62.4
Perceived distance to reach nearby health facility on foot(n=591)		
1h	471	79.7
>1h	120	20.3
Means of transportation(n=591)		
On foot	334	56.5
By Bajaj/Taxi	210	35.5
By Private vehicle	47	8.0
Autonomy to maternal health service (n=591)		
By joint decision	216	36.5
By self-decision	283	47.9
By husband decision	92	15.6
From whom you receive health care access assistance (n=591)		
From my husband	431	72.9
From relatives	111	18.8
From families	31	5.2
From neighbors	18	3.0
Being a model household(n=591)		
Yes	322	54.5
No	269	45.5

### Respondents level of knowledge of PCC

The level of women's knowledge about preconception care was determined by their correct responses to eight knowledge assessment questions, and 269 (45.5%) of those who responded were knowledgeable. Almost seven out of ten (69.4%) of women heard about PCC. More than half of the participants (55.7%) knew at least one behavioral risk factor for a childbearing woman, such as alcohol consumption, cigarette smoking, and illegal drug intake, which could be prevented by having PCC. Nearly half (49.9%) of mothers were aware of the health benefits of receiving PCC. At least one primary message that could be transmitted during PCC was known by 236(39.9%) of respondents (Table4).

**Table 4:** Knowledge of respondents on PCC in Wolkite town, Southern Ethiopia, February 130, 2020

Knowledge assessment variables	Frequency	Percent
Source of information about the PCC (n=591)		
Health professionals at the health center and Hospitals	248	41.9
Health extension workers	288	48.7
Friends	169	28.6
Mass media	142	24.0
No, I didnt hear about PCC	181	30.6
Do you know the direct beneficiaries of PCC? (n=591)		
Only women	198	33.5
Only men	75	12.7
Both men and women	318	53.8
Do you know a place where a woman can get a PCC? (n=591)		
At home level	49	8.3
At health institution	114	19.3
At home and health institution	182	30.7
I dont know	246	41.6
Can you list at least one pregnancy disorder or health issue that can happen but can be minimized by PCC? (n=591)		
Heart diseases including hypertension	99	16.7
Diabetes mellitus	107	18.1
STI/HIV	118	19.9
Stress	32	5.4
I dont know	286	48.4
Can you discuss at least one behavioral risk factor for a childbearing woman that can be avoided by obtaining PCC?		
Alcohol consumption	257	43.4
Cigarette smoking	236	39.9
Illegal drug intake	68	11.5
I dont know	258	43.6
Do you know of at least one intervention or message that can be given during the PCC to a woman? (n=591)		
Folic acid supplementation	166	28.1
Avoiding excess alcohol consumption	86	14.5
Adequate meal frequency	99	16.7
Cessation of cigarette smoking	112	18.9
Free from stress	66	11.1
I dont know	355	60.1
Do you know about PCC service providers? (n=591)		
Health care workers at hospitals and health center	293	49.6
Health extension workers	104	17.6
I dont know	235	39.7

### The practice of preconception care (PCC)

**Fig. 1:**
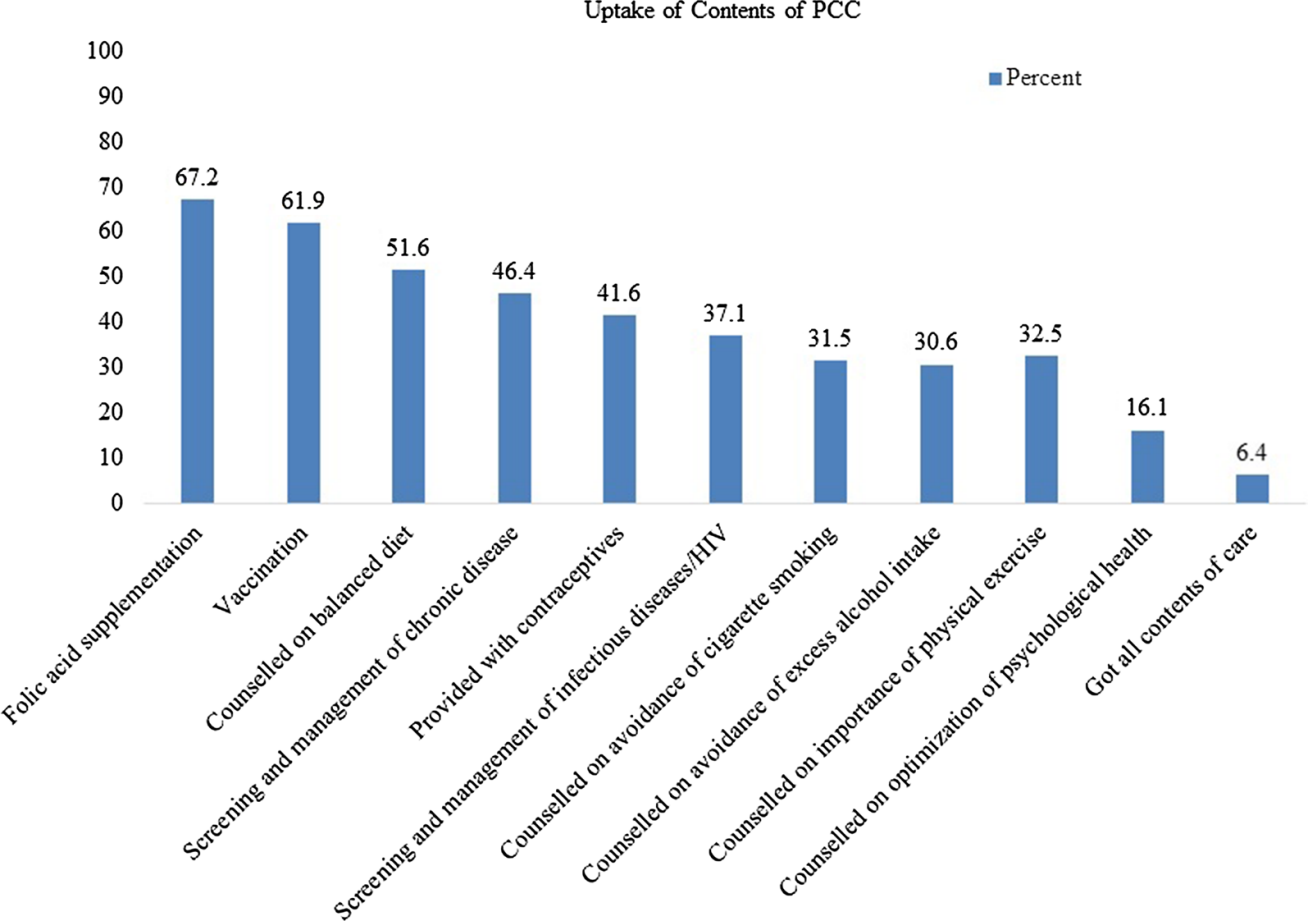
The proportion of women receiving preconception care packages recommended by the World Health Organization in Wolkite town, Southern Ethiopia, 2020 (N = 591)

The current study centered on the ten core elements of PCC services, as previously stated. The mean (SD) score of PCC item uptake was 3.92 (1.98) with minimum and maximum scores of 1 and 10 respectively. Only 6.4% (95% CI 4.2, 8.1) of mothers received all ten selected items of PCC services. Regarding the individual components of care, the most common item received by 67.2% of mothers was folate supplementation, closely followed by vaccination by 61.9% of mothers. The least received element was services focused on improving psychological wellbeing for the coming pregnancy and childbirth (16.1%) (Fig.1). In terms of service delivery points, more than half of the respondents (51.6%) received such services at a health center, followed by 41.3% at a health post, and 7.1% at a hospital.

### Womens experience on PCC

**Fig. 2:**
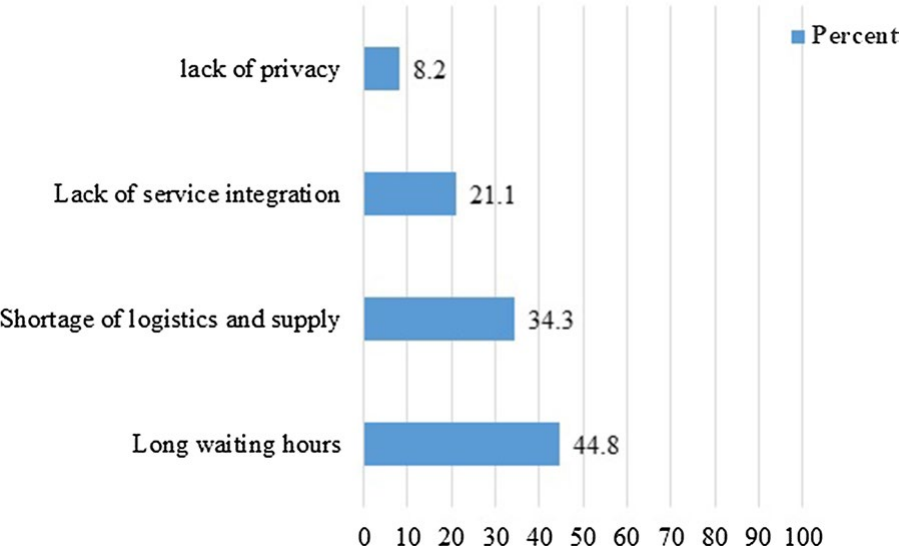
Challenges faced by respondents during receiving PCC in Wolkite town, southern Ethiopia, 2020

Of the total respondents, 397 (67.2%) had prior experience of PCC with at least one item of care, while 194 (32.8%) had no prior experience. Of those experienced respondents, 96 (24.1%) reported that they had faced challenges during receiving care. Consumption of extended time during care provision, 43 (44.8%) and long waiting time to get the health care providers, 31 (32.3%) were the major challenges reported by the respondents (Fig.2). Nearly a fifth, 116 (19.6%) of respondents, had social influence while seeking care, with 69 (59.4%) being influenced by their husband, 44 (37.9%) by close friends and 16 (13.8%) by their families. Just 91 (15.4%) of the study participants did not obtain support from their husbands for PCC, with 56 (61.5%) of the husbands lack awareness about how preconception care benefits couples, 29 (31.8%) mentioning the time spent for care as wastage, and 21 (23.1%) stating a fear of negative attitudes in the future.

### Determinants of the practice of preconception care (PCC)

Results of the multivariable generalized linear regression analysis with a Poisson link identified, Six variables, namely; mother education status, perceived time spent to reach nearby health facilities, availability of PCC unit, mothers knowledge on PCC, being a model household, and having maternal health service autonomy were defined as determinants of the practice of PCC (Table5). The level of education attained by the mother was found to have a significant positive relationship with the receipt of PCC services. Mothers with no formal education were 16% less likely to practice PCC than mothers with a college education or above [AOR 0.84, CI 0.73, 0.97]. Perceived time spent to reach nearby health facilities found as a PCC determinant. Mothers who had to travel more than 30min to get to a health facility were 31% less likely to use PCC [AOR 0.69, CI 0.58, 0.83]. Women's autonomy was significantly associated with their use of the PCC services. Non-autonomous women were 15% less likely than autonomous women to receive PCC services [AOR 0.85, CI 0.75, 0.96]. Furthermore, the availability of a PCC unit was found to be a significant predictor of PCC utilization. The existence of a PCC unit nearby was found to increase the likelihood of PCC service uptake by 46% [AOR1.46; 95%CI 1.17, 1.57]. Maternal knowledge of PCC was also an important predictor of the receipt of PCC items. Respondents with good knowledge of PCC have had a 34% higher chance of utilizing recommended items of PCC service [AOR 1.34, CI 1.13, 1.65]. Compared to respondents who were not from model households (MHHs), those respondents from MHHs had a 31% higher likelihood of receiving PCC services items [AOR 1.31, 95%CI 1.12, 1.45] (Table 5).

**Table 5:** Determinants of the practice of PCC among women of the reproductive age group using a multivariable generalized linear regression model with Poisson link in Wolkite Town, Southern Ethiopia, February 130, 2020

Variables categories	Mean of a component of PCC	p-value	AOR(95%CI)	p-value*
Womens age(N=591)				
35+	3.5	0.000^a^	0.91(0.79,1.04)	0.288
2534	3.8		0.96(0.87,1.05)	0.394
<24	4.2		1	
Womens education level (n=591)				
No formal education	3.5	0.000^a^	0.74(0.63,0.97)	0.009
Primary education	3.7		0.88(0.77,0.99)	0.231
Secondary education	4.1		0.89(0.78,1.11)	0.413
College and above	4.6		1	
Wealth index (n=591)				
Richest	4.1	0.135^a^	1.026(0.89,1.17)	0.549
Rich	4.2		1.06(0.93,1.21)	0.631
Middle	3.9		1.01(0.89,1.150	0.549
Poorer	3.8		0.95(0.83,1.083)	0.569
Poorest	3.6		1	
Family size (n=591)				
<5	4.0	0.115^b^		
5	3.7			
Parity(n=591)				
Grand multiparous	3.7	0.000^a^	0.95(0.84,1.08)	0.608
Multiparous	3.8		0.92(0.83,1.03)	0.402
Primiparous	4.5		1	1
Pre-pregnancy utilization of Contraceptives(n=591)				
No	3.6	0.238^b^		
Yes	4.1			
History of adverse birth outcome (n=591)				
No	3.6	0.001^b^	0.99(0.90,1.09)	
Yes	4.3		1	
Availability of PCC unit(n=591)				
Yes	4.4	0.000^b^	1.46(1.17, 1.57)	<0.001
No	3.5		1	
Perceived time to reach Health facility (n=591)				
Far (>30min)	3.2	0.000^b^	0.69(0.58,0.83)	<0.001
Close (<=30min)	4.1		1	
Autonomy in decision making (n=591)				
By husband and other parties	3.4	0.000^a^	0.85(0.75,0.96)	0.025
By joint decision	3.7		0.94(0.85,1.03)	0.281
By her decision	4.3		1	
Knowledge of PCC (n=591)				
Not knowledgeable	3.3	0.000^b^	1	
Knowledgeable	4.6		1.34(1.16,1.62)	<0.001
Being MHH (n=591)				
No	3.3	0.000^b^	1	
Yes	4.5		1.31(1.18,1.52)	<0.001

Key: 1: reference category

*AOR*adjusted odds ratio

p-value*: indicates p-value at Generalized linear model with Poisson log link

p-values with ^a^ indicates descriptive analysis by using ONE WAY ANOVA

p-value with ^b^ indicates independent t-test analysis

## Discussion

The current study found that only 38(6.4%) of respondents received all the ten WHO recommended items of preconception care services before they underwent their last pregnancy. The mean (SD) score for receiving PCC service items was 3.99(1.99). This suggests that most of the study participants did not adopt the PCC elements suggested by WHO and CDC [1, 7, 47]. The justification for this may be that PCC service provision was given less attention, and the majority of the current focus areas were prenatal care and skilled delivery services. Furthermore, a substantial number of mothers were unaware of PCC programs, which might lead to low uptake. Therefore, town health departments and health care providers must work together to integrate the PCC with routine maternal and child health programs, as well as strengthen awareness-raising efforts to encourage better use of PCC service components.

The most common item received in the study area was micronutrient supplementation (67.2%), which is lower than a report from a similar study conducted in Mekele city, northern Ethiopia (86.3%) [30], but higher than another study conducted in western Ethiopia (7.7%) [32]. The disparity may be due to differences in the study area in which the subsequent study with low prevalence [32] was conducted among rural women. Vaccination was the other item received by the majority of respondents (61.9%) and is comparable to a similar study conducted in Mekelle City, Northern Ethiopia (60.8%) [30], and higher than another study conducted in Western Ethiopia (17.6%) [32]. In the current study area, the adoption of all remaining items of PCC service also requires due emphasis, particularly on the pre-pregnancy contraceptive provision (39.2%), STI screening and management (37.1%), and psychological health optimization (16.1%).

Maternal education, the availability of the PCC unit, maternal knowledge of the PCC, being a model household (MHH), and having maternal health service autonomy were identified as significant determinants of PCC practices.

The mother's educational level was found to have a significant association with the receipt of PCC service items. Similar findings were observed in studies conducted in China, Sri Lanka, Saudi Arabia, and Northern Ethiopia [27,29, 33]. The possible justification could be that women with a higher level of education have more access to information about PCC with strong information-processing skills, leading to the uptake of those recommended contents of care. It is also recognized that education also increases the health-seeking behaviors of mothers by increasing their autonomy and creating greater confidence and ability to make decisions about their health [36, 48].

Also, the current study revealed a significant positive association between maternal knowledge of PCC and the use of PCC service items. Compared to those mothers who were not knowledgeable, the chances of PCC practice were 1.34 times greater for knowledgeable mothers. This result is in line with the studies conducted in China, Saudi Arabia, and northern Ethiopia [29, 31, 49]. This is plausible because the more PCC knowledge a mother has, such as the advantages, content, place, and duration of the visit, the more likely she is to adopt the suggested PCC content. As a result, a concerted effort is needed from the responsible bodies in the town to improve women's knowledge on PCC through behavioral change communication.

The distance (perceived time spent to reach health facilities) was identified as significant predictors of PCC uptake. Mothers who travelled more than 30min to access health facility were 31% less likely to practice PCC than their counterparts. While universal access to the MNCH continuum of care is strongly advocated, there is still a huge difference in the ease of access to health services [50, 51]. This means that to access those mothers who are far from the nearest health facilities, outreach programs should be improved.

The availability of PCC units in a nearby health facility was also correlated with increased odds of using PCCs. Women who mentioned that a preconception care unit is available had a 46% higher chance of using PCC than women who did not know about the existence of a preconception care unit, and this finding is confirmed by a study conducted in Debire Birhan town, northern Ethiopia [30]. This may be that if health facilities had a functioning PCC unit, mothers would have the opportunity to be informed about the importance of getting PCC during their visit, which would enhance PCC uptake. Therefore, a concerted effort from all stakeholders is expected to improve and integrate PCC into the existing MNCH service delivery points.

The autonomy of women in decision-making about their household activities is significantly associated with the practice of PCC in this report. For mothers whose autonomy is at the hands of their husbands or other groups, the likelihood of obtaining a PCC was lower compared to an autonomous one. This research is supported by by studies elsewhere that indicate uptake of maternal health care services is affected by women's positions in decision making [42, 52, 53]. A potential argument may be that there is a greater chance for autonomous women to access reproductive health care services [54]. Also, the control of women over household resources could have a significant positive impact on both the demand for and the uptake of MNCH services [48].

Among mothers who were from model households (MHH), the probability of PCC practice was greater. Those mothers from MHHs were 1.3 times more likely than their counterparts to practice PCC. This could be because, Health Extension Workers (HEWs) spend more time on the capacity building portion for model HHs through intensive training, support, and follow-up and family education on MNCH services for those selected to be role models [55, 56]. In contrast to their counterparts, this successive preparation, support, and follow-up could bring skill development and make them exercise PCC adequately and effectively.

There were both strengths and shortcomings in this research. The study is the first of its kind to assess the determinants of the practice of PCC in this study area. The results of the study may have significant policy implications for the further enhancement of PCC services. Although the necessary efforts have been made to mitigate the potential shortcomings of this study, readers should be careful when interpreting the results. Since the study was based on self-reports, the respondents might be prone to social desirability bias. Finally, because women were asking about incidents that had already occurred during the last one year before this study, there may be a risk of recall bias.

## Conclusion

In the study area, the mean score and overall PCC practice were low. Determinants for PCC practice were recognized for the level of education of mothers, mother's knowledge of PCC, time spent reaching nearby health facilities, availability of PCC units, being a model household, and women's autonomy in decision-making. Therefore, increased efforts are needed by policymakers to improve the autonomy of women in decision-making. By integrating with routine MNCH service delivery points, the Health Unit of the city should focus on the functionality of PCC units. Besides, to increase maternal knowledge on PCC, health workers must give due focus on improving behavioral change communication operations.

## Data Availability

All data are fully available without restriction.

## Abbreviations

AOR: Adjusted odds ratio
CDC: Center for Disease Control
COC: Continuum of Care
PCA: Principal Component Analysis
PCC: Preconception Care
SDG: Sustainable Development Goal
SPSS: Statistical product and service solutions
WHO: World Health Organization
